# Effect of Supplementation of Flour with Fruit Fiber on the Volatile Compound Profile in Bread

**DOI:** 10.3390/s21082812

**Published:** 2021-04-16

**Authors:** Robert Rusinek, Marzena Gawrysiak-Witulska, Aleksander Siger, Anna Oniszczuk, Aneta A. Ptaszyńska, Jarosław Knaga, Urszula Malaga-Toboła, Marek Gancarz

**Affiliations:** 1 Institute of Agrophysics, Polish Academy of Sciences, Doświadczalna 4, 20-290 Lublin, Poland; m.gancarz@ipan.lublin.pl; 2 Institute of Food Technology of Plant Origin, Faculty of Food Science and Nutrition, Poznań University of Life Science, Wojska Polskiego 28, 60-637 Poznań, Poland; wima@up.poznan.pl; 3 Department of Food Biochemistry and Analysis, Faculty of Food Science and Nutrition, Poznań University of Life Sciences, Wojska Polskiego 31, 60-634 Poznań, Poland; aleksander.siger@up.poznan.pl; 4 Department of Inorganic Chemistry, Medical University of Lublin, Chodźki 4a, 20-093 Lublin, Poland; anoniszczuk@o2.pl; 5 Department of Immunobiology, Institute of Biological Sciences, Faculty of Biology and Biotechnology, Maria Curie-Skłodowska University, Akademicka 19, 20-033 Lublin, Poland; aneta.ptaszynska@poczta.umcs.lublin.pl; 6 Faculty of Production and Power Engineering, University of Agriculture in Kraków, Balicka 116B, 30-149 Kraków, Poland; jaroslaw.knaga@ur.krakow.pl (J.K.); umalagatobola@gmail.com (U.M.-T.)

**Keywords:** wheat bread, electronic nose, volatile organic compounds, gas chromatography-mass spectrometry, fruit-origin additives

## Abstract

This paper presents the analyses of the effect of fiber additives on volatile organic compounds in bread. The bread was baked from wheat flour with the addition of 3% of fruit fiber, following common procedures. After baking, volatile organic compounds contained in the control bread and breads supplemented with cranberry, apple, and chokeberry fiber were determined. The SPME/GC-MS technique was used for the identification of the odor profile, and the electronic nose Agrinose (e-nose) was used to assess the intensity of the aroma. The results of the analyses revealed the profile of volatile organic compounds in each experimental variant, which was correlated with responses of the electronic nose. The results indicate that the volatile compound profile depends on the bread additives used and influences the intensity of bread aroma. Moreover, the profile of volatile organic compounds in terms of their amount and type, as well as the intensity of their interaction with the active surface of the electrochemical sensors, was specific exclusively for the additive in each case.

## 1. Introduction

Flavor is one of the most important factors in the interaction between the consumer and the food product [1]. A positive or negative response has an impact on consumer’s choices [2]. Hence, there are many grounds for investigations and analysis of food flavors. Flavor (or taste) is one of the basic senses available to organisms used for the chemical analysis of the composition of food. In many organisms, flavor and smell are not separated. A criterion for separating these senses is the detection of information about a nearby or distant source. The smell is a stimulus with a more dynamic impact on the consumer’s choice via a mechanism of fragrance compounds reaching the human olfactory apparatus without the need for physical contact with the product. Volatile organic compounds (VOCs) constituting the aroma composition can be defined as a family of carbon-containing chemicals exhibiting high vapor pressure at ambient temperature. They are countless, diverse, and ubiquitous in nature and represent numerous groups of organic compounds with molecular masses ranging from 30 to 200 Daltons. These organic gases are emitted from various sources, including food [1], where aromas play a special role in the interaction with the consumer, especially in the case of bread, which is an essential element in human nutrition. Bread products are included in the so-called food pyramid in all healthy diet schemes. As indicated by recent data, they should be part of the daily diet of a healthy subject. The freshness of bread is frequently assessed based on its aroma [2]. Besides freshness, the consumer often associates the aroma with the expected taste of bread. The taste itself is directly associated with the type and species of the grain used and, more frequently, with bread additives [3,4,5]. Supplementation of bread with natural additives, e.g., those of fruit origin and seeds, is appreciated by many consumers [6,7]. They are most often added to bread dough before the baking process. Besides the aroma, such additives considerably modify the color and structure of bread crumbs and crust [5,8,9,10,11]. Additionally, they may influence the storage properties of the product. There are some well-known instrumental and organoleptic aroma evaluation methods. A sensory panel and a consumer panel represent organoleptic methods for the evaluation of aromas. In turn, chromatographic techniques are the best tools among instrumental methods. Another approach is based on a combination of instrumental and sensory techniques, e.g., GC-MS-O (gas chromatography-mass spectrometry coupled with olfactometry) [12,13]. 

The chromatographic technique is a relatively precise method for the identification of volatile compounds. There are hundreds of volatile substances responsible for the composition of fragrance, but usually, only several main compounds present in the largest amounts are the responsible aromas. The so-called electronic nose is used for the analysis of aromas [14]. The device consists of many chemically sensitive sensors detecting the main volatile compounds. In contrast to chromatographic techniques, the electronic nose facilitates analyses of VOCs in real-time; therefore, this device is perfect for screening examinations, detection of specific volatile substances, identification of aromas, and determination of aroma intensity [15,16,17]. 

The aim of the study was to apply chromatographic techniques and the electronic nose to determine the odor profile of bread supplemented with fruit fibers. The results of chromatographic analyses helped to identify the main groups of VOCs involved in the aroma of the bread. 

## 2. Materials and Methods

### 2.1. Bread

The bread was made of winter wheat flour representing the Universum bread variety. The wheat was cultivated in eastern Poland in Lubelskie Province. The 750 g control wheat bread was composed of wheat flour type 750 (520 g), deionized water (370 mL), dry yeast (4.37 g), and salt (5.2 g). The percent composition of the wheat flour was specified by the manufacturer; it included carbohydrate (71%), protein (12%), fiber (2.9%), fat (1.8%), and water (12.3%). The dough was supplemented with three fruit-origin additives: cranberry fiber, chokeberry fiber, and apple fiber (Microstructure, Poland, Warsaw). Cranberry fiber is an excellent source of anthocyanins and catechins. It delays the aging process, regulates fat metabolism, and removes heavy metals and free radicals. Apple fiber enhances the feeling of satiety thus preventing weight gain, supports the work of intestines, protects the organism against toxic substances, and reduces fat and cholesterol absorption. Chokeberry fiber provides health-promoting antioxidants, scavenges free radicals and prevents the generation of new reactive species, delays the aging process, and supports metabolism. In each variant of the experiment, the fibers constituted 3% of the flour mass, except for the control bread. The composition of the fibers used is shown in Table 1.

All stages of bread production (dough kneading, fermentation, bread baking) were performed automatically using a 1600 W commercial bread maker B11-A (Tefal, France, Rumilly, Haute-Savoie) [18]. The flour, yeast, and salt were mixed for 60 s and the mixture was placed in a 5 L cuboid baking mold filled with 370 mL of deionized water. A mold equipped with two bottom stirrers was used for the subsequent bread production processes: kneading (61 min), fermentation (62 min), and baking (62 min). The three stages lasted 185 min in total, as described in our previous work [19]. The internal dough temperature and the temperature of the bread and the oven chamber were recorded during the baking stage [18,20,21].

### 2.2. Electronic Nose and Three-Parameter Method for Generation of Smellprints

The Agrinose e-nose used in the present study was designed and constructed at the Institute of Agrophysics, Polish Academy of Sciences in Lublin. The device is equipped with a matrix of eight MOS (metal oxide semiconductor) sensors: TGS2600 (hydrogen and carbon monoxide, general air contaminants); TGS2602 (ammonia, high sensitivity odorous gases); VOC; TGS2603 (high sensitivity to odors generated from spoiled foods); TGS2610 (high sensitivity to LP gas, butane); TGS2611 (high sensitivity to methane, natural gas); TGS2612 (high sensitivity to propane, methane, and butane); TGS2620 (high sensitivity to volatile vapors, solvent vapors, alcohol); AS-MLV-P2 (volatile vapors CO, hydrogen, butane, ethanol, methane) [2,21]. 

In contrast to the common methods for description of the odor profile, a new three-parameter method for generation of smellprints was used in the present study, and a new aroma intensity factor, i.e., the ratio of the response times T_ratio_ was used (see Supplementary Materials). This parameter was implemented based on a new method developed for the generation of smellprints based on two additional parameters. These are t_R_ (response time), i.e., the time required for the achievement of maximum response, and t_CL_ (cleaning time), which indicates the time of removal of molecules from the sensor’s active surface, i.e., the time from the achievement of the maximum response ΔR/Rmax to half of its value. These parameters depended on the type of volatile substances contained in the odor profile and on the intensity of emission of these compounds. In terms of its physical interpretation, the higher than zero its value is, the more intense the interaction of volatile substances with the active surface is [22,23]. 

The measurement cycle and the sampling protocol consisted of baseline a purge for 10 s, sample draw-in for 60 s, and sample purge for 140 s. The DasyLab software was used to convert analog signals to digital signals. The graph obtained was converted to the ∗.xls format and analyzed using statistical software.

### 2.3. SPME/GC-MS

The Trace GC Ultra gas chromatograph (ThermoFisher Scientific, Waltham, MA, USA) coupled with an ITQ 1100 mass spectrometer (ThermoFisher Scientific, Waltham, MA, USA) according to the procedure described in a previous study [21,24] was used for GC-MS analyses of bread aroma. Volatile compounds were collected from the headspace by solid-phase micro-extraction (SPME). The SPME fiber 50/30 µm Divinylbenzene/Carboxen/Polydimethylsiloxane (DVB/CAR/PDMS), Stableflex (2 cm) 24 Ga (Sigma Aldrich, Poznań, Poland), was used for chromatographic analyses. The fiber was placed for 30 min in the measuring chamber with a mixture of volatile organic compounds emitted from the bread (temperature of 22 °C and relative humidity of approx. 70% in the chamber). Next, the desorption of volatile organic compounds was performed upon transfer into a GC injector for 5 min. The injection port was equipped with a 0.75 mm i.d. liner maintained at 250 °C in the splitless mode. A Zebron ZB-5Msplus Capillary GC (Torrance, CA, USA) 30 m × 0.25 mm × 0.25 μm capillary column was used. The analyses were performed at 60 °C for 5 min (initial temperature), from 60 to 250 °C at 5 °C/min, from 250 to 270 °C at 10 °C/min, and the final temperature was maintained for 5 min. The helium flow rate was constant at 2.2 mL/min. The temperature of the transfer line and ion source was 280 °C. The electron impact ionization (EI+) mode with an electron energy value of 70 eV was applied. The mass spectrometer collected data in the full scan mode in the ranges of 35–390. The procedure was also described by Rusinek et al. 2020 [22].

### 2.4. Chemometrics

The analysis of the main components, variance, and simple correlations was performed at the significance level α = 0.05 with Statistica software (version 12.0, StatSoft Inc., Palo Alto, CA, USA). The principal component analysis was performed to determine the relationship between the sensor response ΔR/Rmax and T_ratio_ for all sensors used in the study and all volatile compounds determined in the three bread additives and the control bread [25]. The optimal number of the principal components obtained in the analysis was determined based on the Cattel criterion. A data matrix with 27 columns and 12 rows was constructed for correlation of the electronic nose results and gas chromatography analysis [26]. The input matrix was scaled automatically.

## 3. Results and Discussion 

### 3.1. Electronic Nose

Table 2 shows the mean values of the ΔR/R_max_ parameter, which should be interpreted as the intensity of the odor profile of the analyzed bread, and the calculated T_ratio_ value, i.e., the dynamics of changes in the measurements of the volatile compounds. The ΔR/R_max_ parameter and the T_ratio_ value were characteristic and specific for each bread sample [1]. One of the highest levels of maximum responses (ΔR/R_max_) was found in the case of the control bread sample, whereas the lowest sensor response signals were recorded for the chokeberry fiber-supplemented bread, whose odor profile, as revealed by the GC-MS analysis, comprised the lowest number of volatile compounds. The T_ratio_ coefficient was reflected in the dynamics of adsorption and desorption of the bread aroma molecules.

The coefficient value in most cases was lower than 1, except for the control bread. This indicates that the purification time was equal to or shorter than the response time, and the molecules were desorbed from the active surface of the sensor more easily than they were adsorbed. Similar observations for chemical standards (3-methyl-1-butanol, ethanol, hexanal, limonene) were reported by Gancarz et al., 2019 [23]. This suggests that the response time and the purification time depend on the odor profile composition and may change with the changing proportions in the profile. Similar studies on the use of metal oxide semiconductor sensors for detection of the odor profile have been conducted by many authors [14], who have analyzed signals to determine the presence of mold on bread or supplementation additives [24]. Similarly, Lippolis et al., (2014) [21] used an electronic nose to classify wheat in terms of fungal infection in the material. In the present study, one of the matrix sensors, i.e., TGS2612, differed in its response to the intensity of the odor profile and generated the lowest response values of changes in the resistance. This is probably associated with the presence of a carbon filter in its structure.

### 3.2. SPME/GC-MS

The analysis of the chromatograms generated for the individual samples allowed identification and classification of volatile compounds contained in the odor profile of the control bread and the bread supplemented with cranberry, apple, and chokeberry fiber into the main chemical groups (Table 3). For the identification of the compounds, the Wiley 138 library was used with the highest quality of matching in the range of 60–95% [25]. Groups containing terpenes, alcohols, ketones, steroids, esters, acids, pyridines, nitriles, phenols, and aromatic hydrocarbons were identified [26]. These groups represented the core compounds present in various proportions in the odor profile [27]. 

The greatest number of these VOCs was detected in the control non-supplemented bread, whereas their lowest number was found in the case of the chokeberry fiber-supplemented bread, which emitted the least intense aroma, as indicated by the organoleptic observations carried out by the authors. A similar relationship was shown with the use of the electronic nose and the ΔR/R_max_ parameter for detection of the intensity of the bread odor profile. In analyses of the aroma of roasted coffee beans from different regions of the world, this parameter was positively correlated with the amount of pyridine, which is responsible for e.g., the bitterness in coffee infusions [22]. 

### 3.3. Principal Component Analysis

**Figure 1:**
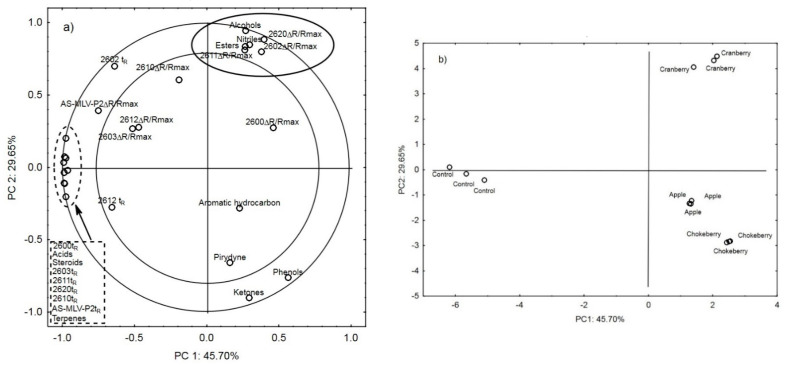
(**a**) Projection of the variables: main chemical compounds and electronic nose responses to the PC1 and PC2 factor plane; (**b**) projection of the cases of supplementing bread with chokeberry fiber, apple fiber, cranberry fiber and for control bread to the PC1 and PC2 factor plane.

Figure 1a shows the projection of the variables (results of the analysis conducted with the electronic nose and GC-MS) on the factor plane, which demonstrates correlations of the individual sensor responses with the quantities of the volatile compound groups [28]. The first two principal components describe 45.70% (PC1) and 29.65% (PC2) of correlations, or 75.35% of the system variability. The first principal component, PC1, differentiates baked bread according to its aroma resulting from apple, chokeberry, and cranberry fiber supplementation from the control bread. As shown in the figure, there is a strong positive correlation of alcohols, nitriles, and esters with the maximum responses of the TGS2611, TGS2620, and TGS2602 sensors and with the dynamics of molecule adsorption and desorption expressed by the T_ratio_ parameter for acids, steroids, terpenes, and the TGS2600, TGS2603, TGS2611, TGS2620, TGS2610, and AS-MLV-P2 sensors.

Figure 1a shows a strong negative correlation between the AS-MLV-P2 ΔR/R_max_ parameter, T_ratio_ for TGS2602, and the content of phenols and ketones in the odor profiles of the bread. Figure 1b presents the projection of cases on the factor plane differentiating the bread odor profile with regard to the additives used. The first principle component PC1 differentiated the samples into the control bread (negative PC1 values) and the vegetable fiber-supplemented bread (positive PC1 values). The level of the response of the TGS2600, TGS2603, TGS2611, TGS2620, TGS2610, and AS-MLV-P2 sensors in combination with the levels of acids, steroids, and terpenes exerted the greatest effect on the differentiation of the control bread from the fruit fiber-supplemented breads (Figure 1a). A similar effect on the differentiation of the odor profile of the cranberry fiber-supplemented bread was exerted by the level of alcohols, nitriles, and esters in combination with the responses of the TGS2611, TGS2620, and TGS2602 sensors. In this case, these relationships were described by the second principal component PC2, more specifically, by its positive values (Figure 1b).

**Figure 2:**
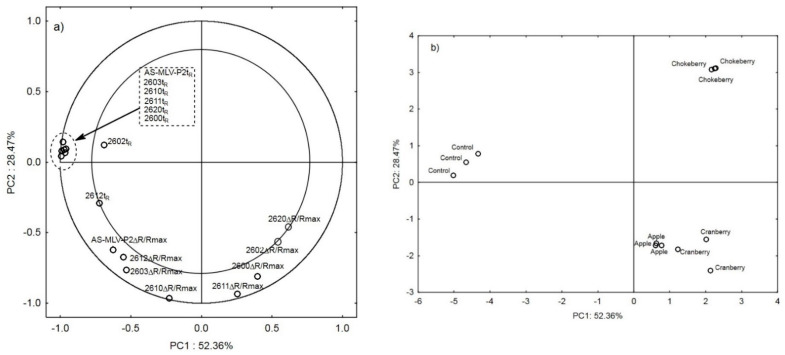
(**a**) Projection of the variables: electronic nose responses to the PC1 and PC2 factor plane; (**b**) projection of the cases of supplementing bread with chokeberry fiber, apple fiber, cranberry fiber, and for control bread to the PC1 and PC2 factor plane.

Similar to Figure 1a, Figure 2a shows the projection of the variables on the factor plane, but only for the electronic nose results [29]. The results surrounded by the dashed-line ellipse represent six cleaning-to-response time ratios, which distinguish the odor profile of the control bread from the profiles of the supplemented breads.

In the analysis performed with the use of the electronic nose to recognize the odor profile intensity in the breads supplemented with various plant additives [24], the first two principal components describe the relationships in 80.83% of cases. The first principal component PC1 describes the difference between the odor profile of the control bread and the fruit fiber-supplemented breads in 52.36% of cases. The second principal component PC2 describes (in 28.47% of cases) the intensity of the aroma between the breads supplemented with the fruit fibers. The breads with the more intense odor profile and the apple and cranberry additives (higher ΔR/R_max_ signals (Table 1) and a greater amount of volatile compounds determined with the GC-MS technique (Table 2)) are located on the negative side of PC2, whereas the chokeberry profile with the poorer composition of VOCs is located on the positive side of PC2. To sum up, the use of the e-nose technique for detection of the intensity of odor profiles in bread supplemented with plant fiber facilitates identification and assignment of the odor profile to a specific fiber used in the bread baking process [30,31].

## 4. Conclusions

The chromatographic technique employed in the study facilitated the determination of the main volatile compounds constituting the odor profile in breads with and without fiber additives. The amount of VOCs varied depending on the supplement used. More VOCs were determined for the control bread than for the supplemented bread, which may indicate that the supplementation generates other dominant volatile compounds. The electronic nose helped to determine the intensity of the smell expressed by the maximum response of chemically sensitive sensors. Similarly, the dynamics of the interaction of the VOCs with the active sensor surface varied depending on the fiber supplements used in the baking process. The investigations allow concluding that the techniques of detection of VOCs are promising tools for classifying bread in terms of the odor profile, which is one of the most important features taken into account by the consumer in the choice of this product. In the future, an electronic nose can be used as a tool for controlling and distinguishing the supplemented bread.

## Figures and Tables

**Table 1:** Nutritional value-composition of basic nutrients in fruit-origin additives (fruit fibers).

Nutritional Value	Cranberry Fiber	Chokeberry Fiber	Apple Fiber
Energy value	18.24 kcal	17.35 kcal	45.14 kcal
Fat	0.53 g	0.57 g	0.6 g
Including saturated fatty acids	0.065 g	0.069 g	0.075 g
Carbohydrates	2.74 g	2.47 g	4.51 g
Including sugars	≤0.1	≤0.1	≤0.2
Soluble fiber	1.9 g	1.85 g	1.85 g
Insoluble fiber	9.39 g	9.7 g	7.47 g
Protein	1.02 g	0.91 g	1.11 g
Salt	0	0	0

**Table 2:** Mean values of ΔR/Rmax and Tratio with standard deviations.

Supplement	TGS2602	AS-MLV-P2	TGS2603	TGS2612	TGS2610	TGS2611	TGS2620	TGS2600
	ΔR/Rmax							
Control bread	1.23 ± 0.26	3.06 ± 0.05	3.23 ± 0.03	0.1 ± 0.01	0.86 ± 0.05	0.47 ± 0.06	0.7 ± 0.1	0.71 ± 0.01
Bread with cranberry fiber	3.0 ± 0.1	2.4 ± 0.1	2.6 ± 0.3	0.1 ± 0.01	1.01 ± 0.08	0.9 ± 0.01	2.4 ± 0.03	2.41 ± 0.01
Bread with apple fiber	1.52 ± 0.02	1.3 ± 0.1	3.65 ± 0.1	0.1 ± 0.01	1.07 ± 0.01	0.72 ± 0.02	0.73 ± 0.03	3.5 ± 0.1
Bread with chokeberry fiber	1.33 ± 0.26	1.9 ± 0.1	1.2 ± 0.01	0.1 ± 0.01	0.33 ± 0.02	0.33 ± 0.03	0.92 ± 0.02	0.81 ± 0.01
	T_ratio_							
Control bread	0.6 ± 0.1	7.1 ± 0.1	4.5 ± 0.1	0.01 ± 0	1.1 ± 0.1	1.7 ± 0.05	1.1 ± 0.1	1.1 ± 0.1
Bread with cranberry fiber	0.55 ± 0.05	1 ± 0.01	0.53 ± 0.04	0.01 ± 0	0.01 ± 0	0.1 ± 0.01	0.2 ± 0.01	0.3 ± 0.01
Bread with apple fiber	0.41 ± 0.01	0.33 ± 0.03	0.31 ± 0.01	0.01 ± 0	0.29 ± 0.01	0.41 ± 0.01	0.41 ± 0.01	0.2 ± 0.01
Bread with chokeberry fiber	0.21 ± 0.01	0.15 ± 0.01	0.21 ± 0.01	0.01 ± 0	0.15 ± 0.01	0.3 ± 0.03	0.21 ± 0.01	0.21 ± 0.01

**Table 3:** Main VOCs identified in the bread odorant SPME extract.

Peak Number	R_t_	Control Bread	R_t_	Cranberry Fiber	R_t_	Apple Fiber	R_t_	Chokeberry Fiber
1	1.88	4h-1-benzothiopyran-4-one,3-[(2-hydroxyphenyl)amino]-,1-oxide C15H11NO3S Terpene	0.98	1,2-dicyano-3-phenyl-1,2-cyclopropanedicarboxamide C13H10N4O2 Terpene	9.48	2-(2,4-dimethoxy-phenyl)-7-methyl-2,3,5,6,7,8-hexahydro-1H-benzo[4,5]thieno[2,3-D]pyrimidin-4-one C19H22N2O3S Ketones	17.26	4,5,6,7-tetrachydroxy-1,8,8,9-tetramethyl-8,9-dihydro-3h-phenaleno[1,2–β]-3-one C19H18O6 Ketone
2	6.99	[2,8-dimethyl-2-(4,8,12-trimethyltridecyl)-3,4-dihydrochromen-6-yl] (E)-3-[2-(1,2-dihydroxyethyl)-4,5-dioxooxolan-3-yl]oxyprop-2-enoate C36H54O9 Alcohol	2.13	1,2-bis(trimethylosilyloxy)-4-trimethylsilyloxymethylbenzene C16H32O3Si3 Nitrile	22.43	4h-1-benzopyran-4-one,2-(3,4-dimethoxyphenyl)-6,8-di-β-D-glucopyranosyl-5,7-dihydroxy C27H30O16 Ketone	22.54	2-anthracenecarboxylic acid, 9,10-dihydro-3,6,8-trimethoxy-1-methyl-9,10-dioxo-, ethyl ester C21H20O7 Ester
3	11.07	4h-1-benzopyran-4-one,2-(3,4-dimethoxyphenyl)-5-hydroxy-3,6,7-trimethoxy C20H20O8 Ketone	4.42	10.69% x3 acetonitrile C2H3N Nitrile	27.92	pregn-4-ene-3,20-dione,11,17,21-trihydroxy-,(11β) C21H30O5 Ketone	27.06	bufa-20,22-dienolide,3,5,14,-trihydroxy-(3β,5β)- C24H34O5 Terpene
4	12.45	pregn-4-ene-3,20-dione,11,12-dihydroxy-,(11β)- C21H30O4 Ketone	6.93	4-bromo-N-[(6-methyl-2-pyridyl)aminomethyl]phthalimide C15H12BrN3O2 Terpene	34.17	naphtho[1,8-CD]-1,2-ditellurole C10H6Te2 Fenol	34.17	naphtho[1,8-C,D]-1,2-ditellurole C10H6Te2 Fenol
5	13.33	[2-[(6S,8S,9S,10R,11S,13S,14S,17R)-11,17-dihydroxy-6,10,13-trimethyl-3-oxo-7,8,9,11,12,14,15,16-octahydro-6H-cyclopenta[a]phenanthren-17-yl]-2-oxoethyl] acetate C24H32O6 Steroid	11.14	dodecanoic acid,2,3-bis(acetyloxy)propyl ester C19H34O6 Ester	36.16	2,4,6-triselenatricyclo[3.3.1.1(3.7)decan-8-one, 1,3,5,7-tetramethyl- C11H16Ose3 Ketone	34.77	4h-1-benzopyran-4-one,2-(3,4-dimethoxyphenyl)-5-hydroxy-3,6,7-trimethoxy- C20H20O8 Ketones
6	18.67	3-[[3-acetyl-2,4,6-trihydroxy-5-(3-methylbut-2-enyl)phenyl]methyl]-6-ethyl-4-hydroxy-5-methylpyran-2-one C22H26O7 Ketone	17.42	9,12,15-octadecatrienoic acid,2-[(trimetthylsilyl)oxy]-1-[[(trimethylsilyl)oxy]methyl]ethyl ester,(Z,Z,Z) C27H52O4Si2 Ester	40.00	(4Z)-1,1,1-trifluoro-4-[(2-([(E)-4,4,4-trifluoro-1-methyl-3-oxobutylidene]amino)phenyl)imino]-2-pentanone C16H14F6N2O2 Ketone	37.03	cholest-5-ene-16,22-dione,3b,26-dihydroxy-,3-acetate, (20S,25R)- C29H44O5 Ketones
7	22.30	4Aα,4Bβ-gibbane-1α,10β-dicarboxylic acid,4a-formyl-2β,7-dihydroxy-1-methyl-8-methylene-,dimethy ester C22H30O7 Ester	22.50	9-octadecatrienoic acid,(2-phenyl-1,3-dioxolan-4-yl)methyl ester C28H44O4 Ester	47.38	1,2,5-trichloro-4-methoxy-3-(2,3,5-trichloro-6-methoxybenzyl)benzene C15H10Cl6O2 Aromatic hydrocarbon	47.33	2,4-dimethyl-6-(phenylamino)-1h,2h-phthalazino[2′,1′,3,4]pyrymido[4,5-d]pyrimidine-1,3-(2h,4h)-dione C21H18N6O2 Pyrydine
8	23.21	4-hydroxy-4-androstene-3,17-dione glucuronide C25H34O9 Terpene	26.61	β-D-glucopyranoside,metyl 2,3-bis-O-(trimethylsilyl)-,cyclic methylboronate C14H31BO6Si2 Ester	49.86	4h-1-benzopyran-4-one,2-(3,4-dimethoxyphenyl)-6,8-di-β-D-glucopyranosyl-5,7-dihydroxy C27H30O16 Ketone		
9	26.92	2,4-dimethyl-6-(phenylamino)-1h,2h-phthalazino[2′,1′,3,4]pyrymido[4,5-d]pyrimidine-1,3-(2h,4h)-dione C21H18N6O2 Terpene	27.29	curan-17-oic acid,2,16-didehydro-19-hydroxy-,methyl ester,(20,XI)- C20H24N2O3 Ester				
10	29.25	alstozine n-oxide C22H28N2O5 Terpene	29.27	6-amino-5-cyano-4-(2-furyl)-2-methyl-4H-pyran-3-carboxylate C15H14N2O4 Ester				
11	30.90	bufa-20,22-dienolide,3,14-dihydroxy-(3β,5β)- C24H34O4 Terpene	33.52	oxiranecarboxamide,2-ethyl-3-propyl- C8H15NO2 Terpen				
12	37.07	Picras-3-ene-2,16-dione, 13,20-epoxy-1,11,12-trihydroxy-15-(2-methyl-1-oxobutoxy)-, (11beta)- C25H34O9 Ketones	37.13	9,12,15-octadecatrienoic acid,2-[(trimetthylsilyl)oxy]-1-[[(trimethylsilyl)oxy]methyl]ethyl ester,(Z,Z,Z) C27H52O4Si2 Ester				
13	39.93	hydrocortisone acetate C23H32O6 Ester	42.64	8-azabicyclo[3,2,1]octane-2-carboxylic acid,3-(benzoyloxy)-8-methyl-,[1R-(exo,exo)]- C16H19NO4 Ketone				
14	42.37	Picrasan-21-oic acid, 13,20-epoxy-3,11,12-trihydroxy-15-(3-methyl-1-oxobutoxy)-2,16-dioxo-, methyl ester, (11beta,12alpha,15beta)- C26H36O11 Ester	45.80	1,2,3,4,5,6-cyclohexanehexone hexaoxime C6H6N6O6 Terpene				
15	45.00	limonoic acid, di-delta-lactone, mixture with (R)-1-methyl-4-(1-methylethenyl)cyclohexene C36H46O8 Acid						
16	47.29	1h-pyrrole-3,4-dicarboxylic acid,2-(3,5-dichloro-2-methoxyphenyl) diethyl ester C17H17Cl2NO5 Ester						
17	50.08	3,3′,5,3″-bis(dimethylene)-2,6-di(1′,8′-naphthyrid-2′-yl)pyridine C25H17N5 Pirydyne						

R_t_—retention time.

## Data Availability

Not applicable.

## Supplementary Materials

The following are available online at https://www.mdpi.com/article/10.3390/s21082812/s1, Figure S1: Scheme of a typical sensorgram for MOS sensors with ratio of reaction times of t_R_ and t_CL_, marked on the graph.
