# Supplementary material for: Effect of Supplementation of Flour with Fruit Fiber on the Volatile Compound Profile in Bread
Source: Sensors (Basel). 2021 Apr 16;21(8):2812. doi: 10.3390/s21082812 (PMC8073101; doi:10.3390/s21082812)
Supplement: Supplementary file 1 [file sensors-21-02812-s001.zip › sensors-1179484-supplementary.pdf]

Supplementary Materials

Effect of Supplementation of Flour with Fruit Fiber on the Volatile Compound Profile in Bread

Robert Rusinek [1,*], Marzena Gawrysiak-Witulska [2], Aleksander Siger [3], Anna Oniszczuk [4], Aneta A. Ptaszyńska [5], Jarosław Knaga [6], Urszula Malaga-Toboła [6] and Marek Gancarz [1,6]

[1] Institute of Agrophysics, Polish Academy of Sciences, Doświadczalna 4, 20-290 Lublin, Poland; m.gancarz@ipan.lublin.pl
[2] Institute of Food Technology of Plant Origin, Faculty of Food Science and Nutrition, Poznań University of Life Science, Wojska Polskiego 28, 60-637 Poznań, Poland; wima@up.poznan.pl
[3] Department of Food Biochemistry and Analysis, Faculty of Food Science and Nutrition, Poznań University of Life Sciences, Wojska Polskiego 31, 60-634 Poznań, Poland; aleksander.siger@up.poznan.pl
[4] Department of Inorganic Chemistry, Medical University of Lublin, Chodźki 4a, 20-093 Lublin, Poland; anoniszczuk@o2.pl
[5] Department of Immunobiology, Institute of Biological Sciences, Faculty of Biology and Biotechnology, Maria Curie-Skłodowska University, Akademicka 19, 20-033 Lublin, Poland; aneta.ptaszynska@poczta.umcs.lublin.pl
[6] Faculty of Production and Power Engineering, University of Agriculture in Kraków, Balicka 116B, 30-149 Kraków, Poland; jaroslaw.knaga@ur.krakow.pl (J.K.); umalagatobola@gmail.com (U.M.-T.)
* Correspondence: r.rusinek@ipan.lublin.pl; Tel.: +48-81-744-50-61; Fax: +48-81-744-50-67

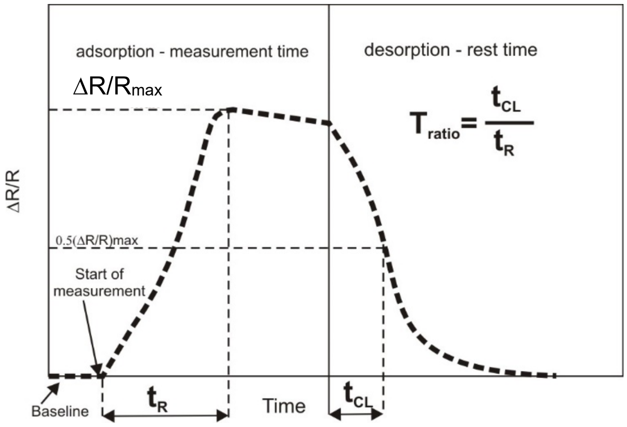

Figure S1. Scheme of a typical sensorgram for MOS sensors with ratio of reaction times of t_R and t_{CL}, marked on the graph.
